# Use of benzodiazepines and detoxification with methadone

**Published:** 2014

**Authors:** G Popescu, C Negrei, D Bălălău, AM Ciobanu, D Baconi, C Bălălău

**Affiliations:** * Department of Toxicology, “Carol Davila” University of Medicine and Pharmacy, Faculty of Pharmacy, Bucharest,; ** Department of Drug Control, “Carol Davila” University of Medicine and Pharmacy, Faculty of Pharmacy, Bucharest,; *** “Sf. Pantelimon” Emergency Hospital, Surgical Clinic, “Carol Davila” University of Medicine and Pharmacy, Faculty of General Medicine

**Keywords:** benzodiazepines, methadone, abuse

## Abstract

**Rationale:** Benzodiazepines are used as anti anxiety drugs, as well as in adjunct treatment for a range of neurological and psychiatric disorders. Abusive patterns of use were increasingly reported and building evidence points to prevalence of benzodiazepines abuse, on one hand as well as to their common abuse in combination with other drugs such as opioids, most frequently.

**Objective:** The main objective of this research is to conduct a systematic study on the behavior profile of a patient admitted to a prison hospital, who is a benzodiazepines user consecutive to admission into a methadone administration program.

**Methods and results:** Statistic values have been taken into account describing the distribution and the distribution form of the various variables studied to find the normality degree of distributions, regarding three measurements at the three moments: before the administration of methadone, immediately after its completion and two months after completion.

**Conclusions and discussions:** The statistic results obtained speak of a strong positive correlation, allowing the support of the fact that persons diagnosed with prescribed/ unprescribed benzodiazepine, use the display association with the admission into a methadone administration program, based on the assumption which concerns a significant positive association between the use of reported benzodiazepine and the administration of methadone in the questioned patients on admission.

As far as the second premise regarding the administration of methadone is concerned it brings about an improvement in the level of benzodiazepines used in research patients, which one may assert that, according to the results obtained, the initiation of methadone therapy in the detoxification program is conducive to the reduction of benzodiazepines use.

## Introduction

Psychotropic drugs are commonly prescribed in the entire world as effective agents within therapies against anxiety disorders as well as in the adjunct treatment for a range of neurological and psychiatric disorders, benzodiazepines (BZD) are characterized by a central chemical structure resulted from the fusion of a benzene and the diazepine ring. Their effect is due to their role as positive allosteric modulators acting on the GABA A receptor complex and their action basically consists of increasing GABA effects by enhancing the flux of chloride (Cl−) and the channel opening rate [**1**-**4**].

As far as the mechanism of their action is concerned, this consists of an activation of the GABA/ steroid/ barbiturate receptor sites, resulting in muscle relaxation. The additional sedative and anxiolytic effect of benzodiazepines has been further explained by the stimulation of the different GABA A subunits. One other effect of BZDs such as diazepam, flurazepam, lorazepam, chlordiazepoxide, midazolam and medazepam is its action as reinforcer, shown in experiments carried out on rodents and non-human primates [**5**-**11**]. The respective reinforcing effect cannot be readily traced back to a single receptor subtype only. This reinforcing effect has been confirmed in humans as well, laboratory BZD studies undertaken having demonstrated their actual capacity to allow self-administration. However, if compared to other drugs such as cocaine, opioids or amphetamines, BZDs’ reinforcing effect is relatively weak [**12**-**14**]. 

Their less than potent self-administration response is nevertheless balanced by their relative accessibility, which, added to their positive subjective effects on the individual has vastly contributed to the abuse potential of the BZD class of drugs [**15**,**16**].

Debate concerning the prevailing nature of BZD abuse has been ongoing: recreational, non-medical or medically adjunctive (associated with the therapeutic use of BZD) [**17**-**21**]. As clinical use of allosteric modulators and GABA A agonists was extending, abusive patterns of use were increasingly reported and building evidence points to the prevalence of BZD abuse, on one hand, as well as to their common abuse in combination with other drugs such as opioids, most frequently. 

## Materials and Methods

Objectives

The main objective of this research was to conduct a systematic study on the behavior profile of a patient admitted to a prison hospital, who was a benzodiazepines user consecutive to the admission into a methadone administration program, providing a theoretically and scientifically integrated view of the evolution of this issue through a specialized approach.

A second objective, transcending the descriptive limits, concerns the identification of these patients’ toxicomedical status based on a number of statistical methods allowing the evaluation of associations between the use of benzodiazepines and methadone with predictive equations indicative of the significance of these statistical trends and the applicability of results within patient samples associated with various somatic and psychosomatic diseases or serious mental disorders.

Research premises

Premise 1. Persons diagnosed with prescribed/ unprescribed benzodiazepine use display association with admission into a methadone administration program.

Premise 2. The administration of methadone brings about an improvement in the level of benzodiazepines use in research patients.

This research has been conducted on a single patients group, at various moments:

1. Prior to the admission into the methadone administration program. The initial group included 84 subjects aged 17-60 years, 73 males and 11 females. 

Patients belong to various professional media, such as: pre-college and college students, unemployed, higher education, retired for health grounds. 

The background interview also involved gathering data on additional dependencies such as alcohol consumption (72 subjects declared daily use); smoking (39 subjects had been smoking for at least two years); drug consumption (all patients admitted consumption at various levels). All subjects volunteered participation in the research, they signed the informed consent and none received ulterior financial compensation. 

2. One month after the admission in the methadone administration program. The group assessed consisted of 55 patients aged 17-55 years, continuing with the study.

The group was made up of patients remaining in the methadone administration program to its completion. 

3. The follow-up, carried out two months after the completion of the methadone administration program with support from healthcare professionals in hospital.

The tools used for data selection were administered individually on the entire group of patients.

Three measurements were conducted for the entire research for each tool at the three moments indicated: before the administration of methadone, immediately after its completion and two months after completion.

On each application, subjects were required to provide both verbal and written responses whereas the application of the interview and scales was accompanied by the gathering and interpretation of specialized biological samples. After having been explained, there were no correct or incorrect responses, all patients were warranted confidentiality of their particulars as well as anonymity of responses.

The interpretation of results was achieved with descriptive indicators such as average, median, manner and standard bias, skewness and kurtosis.

The significance analyses have also been conducted by means of the T-student test as well as criterion prediction tests.

Results thereof have been included in a table and processed according to the SPSS 21.0 program. Descriptive statistic was used to provide synthetic expression of variables considered by means of numeric values characteristic to the respective data. Statistic values have been taken into account describing the distribution and the distribution form of the various variables studied to find the normality degree of distributions.

## Results

Premise 1. Persons diagnosed with prescribed/ unprescribed benzodiazepine, use display association with admission into a methadone administration program.

To study the relationship between the degree of benzodiazepine use and methadone administration in investigated patients, the Pearson correlation coefficient was calculated for the entire hospitalized group. 

Values of correlation coefficients are shown in the tables below as matrices, where each cell includes information on the value of the Pearson correlation coefficient (r), the significance threshold level (p – sig. 2-tailed) and the number of subjects (N).

**Table 1:** Correlations benzodiazepines consumption and methadone administration

	**Correlations**		
		Total BZN	Total Methadone
Total benzodiazepines consumption	Pearson Correlation	1	,602**
	Sig. (2-tailed)		,001
	N	55	55
Total methadone treatment	Pearson Correlation	,602**	1
	Sig. (2-tailed)	,001	
	N	55	55

For the investigated patient group, significant correlations have been obtained between variables: 

- Research group: benzodiazepine use indicated and methadone administration on admission, r(55)= 0,602; p=0,001 < 0,01 bilateral.

In addition, a correlation analysis has been conducted on data obtained from research subjects one month after the treatment initiation stage and follow-up, and at the two months stage, also providing positive correlations characterized by high significance between the two variables.

Post methadone treatment: rpost-treatment 1 month (55) = 0,621; p=0,000 < 0,01 bilateral 

Follow up: rfollow-up 2 months (55) = 0,568; p=0,001 *lt; 0,01 bilateral,

Indicative of the fact that, two months after the treatment, the subjects group preserved significant average correlations between the two variables, slightly lower than on admission. 

Premise 2. Administration of methadone brings about an improvement in the level of benzodiazepines use in research patients.

To test this premise, a variance test is to be performed with repeated mixed type ANOVA-MR measures. This analysis verifies whether the independent variable, admission to the methadone administration program under specialist hospital supervision, has a significant effect on the dependent variable, on-prescription/ non-prescription benzodiazepine use,

**Table 2:** The analysis of multivariate tests

			Multivariate tests c					
Effect		Value	F	Hypothesis grade df	Error df	Sig.	Partial Eta Index	Observed powerb
Stages	Pillai's Trace	,976	1723,604a	2,000	60,000	,000	,981	1,000
	Wilks' Lambda	,017	1723,604a	2,000	60,000	,000	,981	1,000
	Roy's Largest Root	57,520	1723,604a	2,000	60,000	,000	,981	1,000
	Pillai's Trace	,700	70,011a	2,000	60,000	,000	,700	1,000
	Wilks' Lambda	,300	70,011a	2,000	60,000	,000	,700	1,000
	Roy's Largest Root	2,334	70,011a	2,000	60,000	,000	,700	1,000

The multivariate tests table shows that the Pillai’s Trace test has a significant value for the variation of averages of the dependent variable depending on the “Stages” intra-subject factor, as well as a significant value for the Stages* group combination.

The analysis of multivariate tests shows that the methadone therapy effect on hospitalized patients, assessed at the three research stages has a significant effect on the level of benzodiazepine use (Pillai’s Trace= 0,700, F(2, 52)=70,011, p=0,000, η2 partial = 0,976, observed power = 1). 

Secondly, the analysis of the combined effect of the Stages and Group variables shows significant differences between the two groups with regard to methadone therapy effect on BZD use (Pillai’s Trace= 0,976, F(2, 52)=1723,604, p=0,000, η2 partial = 0,70, observed power = 1).

Judging from the analysis of the effect size index, η2 partial = 0,976, methadone therapy may be considered of very high impact on results of the BZD use index (the dependent variable). In this context, the effect size index shows that 97,6% of the dependent variable variance is due to the effect of the independent variable, i.e. 97,6% of the results obtained from measuring the BZD use index are due to effects of the methadone therapy program on patients.

## Conclusions and Discussions

Therefore, it may be maintained that statistic results obtained speak of a strong positive correlation, thus allowing for the support of the first premise of research, based on the assumption concerning a significant positive association between reported BZD use and the administration of methadone in the questioned patients on admission. 

As far as the second premise is concerned, one may assert that, according to the results obtained and presented above, the initiation of methadone therapy in the detoxification program is conducive to the reduction of BZD use.
